# A marine bio-functional lipid, fucoxanthinol, attenuates human colorectal cancer stem-like cell tumorigenicity and sphere formation

**DOI:** 10.3164/jcbn.16-112

**Published:** 2017-05-16

**Authors:** Masaru Terasaki, Hayato Maeda, Kazuo Miyashita, Takuji Tanaka, Shingo Miyamoto, Michihiro Mutoh

**Affiliations:** 1 School of Pharmaceutical Sciences, Health Sciences University of Hokkaido, 1757 Kanazawa, Ishikari-Tobetsu, Hokkaido 061-0293, Japan; 2 Faculty of Agriculture and Life Science, Hirosaki University, 3 Bunkyo-cho, Hirosaki, Aomori 036-8561, Japan; 3 Laboratory of Biofunctional Material Chemistry, Division of Marine Bioscience, Graduate School of Fisheries Sciences, Hokkaido University, Hakodate, Hokkaido 041-8611, Japan; 4 Department of Diagnostic Pathology and Research Center of Diagnostic Pathology, Gifu Municipal Hospital, 7-1 Kashima-cho, Gifu 500-8513, Japan; 5 Epidemiology and Preventions Group, Center for Public Health Sciences, National Cancer Center, 5-1-1 Tsukiji, Chuo-ku, Tokyo 104-0045, Japan

**Keywords:** fucoxanthin, fucoxanthinol, carotenoid, colorectal cancer stem cell, colonosphere

## Abstract

Fucoxanthinol (FuOH), an intestinal metabolite form of fucoxanthin (Fx) isolated from marine algae, is known to possess multiple health benefits, such as prevention of human cancer. However, there is little available information about the effects of FuOH on colorectal cancer stem cells (CCSCs) and their contribution to drug resistance, tumorigenesis and cancer recurrence. In the present study, we investigated the anti-proliferative effect of FuOH on two putative CCSCs, CD44^high^/EpCAM^high^ cells and colonospheres (Csps) formed by HT-29 human colorectal cancer cells, and the suppressive effects of FuOH on the growth of xenografted tumor. FuOH significantly inhibited the growth of CD44^high^/EpCAM^high^ cells and disintegrated Csps and induced many condensed chromatin bodies in the cells in a dose-dependent manner. The IC_50_ value of FuOH for these changes in Csps was 1.8 µM. FuOH down-regulated pAkt (Ser^473^), PPARβ/δ and PPARγ in Csps. These proteins play a critical role in cell proliferation, the cell cycle, metastasis and extracellular adhesion. Ten days after the administration of FuOH (5 mg/kg body weight) to the mice every 3 to 4 days significantly suppressed the Csps tumorigenesis when compared to the untreated control mice. Our results suggest that FuOH could be used as a chemopreventive agent against human CCSC.

## Introduction

**Fig. 1:**
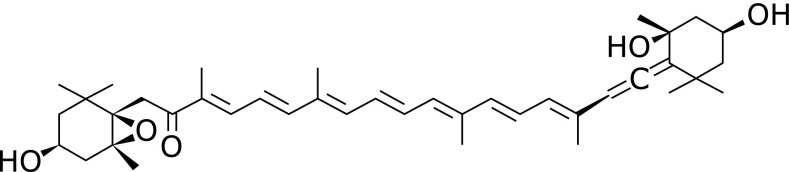
Chemical structure of fucoxanthinol (FuOH). MW: 616.87.

Fucoxanthin (Fx) is an abundant marine xanthophyll with an unusual allenic bond and 5,6-monoepoxide. Fx binds the chlorophyll-protein complex and contributes to efficient light harvesting and photoprotection in marine algae and diatoms. Brown algae, including *Undaria pinnatifida* (wakame), *Hizikia fusiforme* (hiziki) and *Sargassum horneri* (akamoku), which are consumed particularly in Asian countries, are good sources of Fx.^(1,2)^ Wakame consumption per Japanese household has been estimated to be 900 g wet weight/year.^(3)^ Fx content in wakame changes seasonally, with a range of 1.0 to 3.0 mg/g dry weight.^(4)^ Recent accumulating evidence from animal toxicity studies revealed that Fx is a safe compound and induces no adverse effects.^(5,6)^ Fx has multiple beneficial properties, including cancer prevention,^(7–9)^ anti-inflammation and anti-obesity effects.^(10–12)^ Moreover, the anti-cancer effects of Fx have been demonstrated using cultured human cancer cells.^(13–19)^ Fucoxanthinol (FuOH), which is an intestinal metabolite form of Fx, is present as a metabolite in human blood when fed brown algae (Fig. 1).^(20,21)^ Both Fx and FuOH showed strong anti-proliferative effects on cancer cells isolated from the colorectal cancer (CRC) tissue.^(22)^ However, the underlying mechanisms of the anti-cancer activity of Fx and FuOH remain elusive.

CRC is one of the most prevalent human malignancies worldwide.^(23)^ The risk factors for CRC include high intake of animal fat, low intake of vegetables and resultant obesity.^(24)^ Therefore, many preventive trials for CRC have been designed to modify dietary habits but have failed to show efficacy.^(25)^ To date, carotenoids are evaluated as “insufficient” for CRC prevention.^(25)^ However, there are no CRC prospective trials or follow-back studies using Fx or Fx-rich brown algae to determine whether carotenoids are “insufficient” or “sufficient” or “no effects” for CRC prevention.

Colorectal cancer stem cells (CCSCs) that occasionally develop in colorectal tissue may be the start of colorectal carcinogenesis and play a central role in tumor development. CCSCs have several biological properties, including self-renewal, multi-potential, drug-resistance, sphere formation, and tumor formation in xenograft models.^(26,27)^ CCSCs are identified by detecting surface markers, such as CD44, CD133, CD166, EpCAM and Lgr5.^(26,27)^ CD44^+^/EpCAM^high^ cells are considered to be one of the proximate cells displaying the CCSC phenotype.^(26)^ In addition, sphere-forming cells from CRC cells, called colonospheres (Csps), possess CCSC-like properties.^(28)^ Thus, we presume that natural compounds that can induce apoptosis in CCSCs could be a desirable chemopreventive agent against CRC.

In the present study, we investigated the anti-proliferative effects of FuOH on CD44^high^/EpCAM^high^ cells isolated from human CRC cells, HT-29. The molecular mechanisms by which FuOH exerted the effects were investigated. In addition we also examined the effects of FuOH on the tumorigenesis of Csps from HT-29 in the severe combined immunodeficiency (SCID) mice.

## Materials and Methods

### Chemicals and cell culture

All-*trans*-FuOH (purity, ≥98%) was kindly donated by Dr. Hayato Maeda (Hirosaki University, Japan) (Fig. 1). EGF, bFGF and DMEM/F12 medium were purchased from Wako Pure Chem. (Osaka, Japan). B27 was obtained from Miltenyi Biotec, Inc. (Auburn, CA). HT-29 human CRC cells were purchased from American Type Culture Collection (Manassas, VA). These cells were cultured in Dulbecco’s modified Eagle’s medium (DMEM) supplemented with 10% heat-inactivated fetal bovine serum (FBS), 4 mM l-glutamine, 40,000 U/L penicillin, and 40 mg/L streptomycin. The cells were cultured at 37°C in a humidified atmosphere of 95% air and 5% CO_2_.

### Cell isolation and proliferation

HT-29 cells were trypsinized, washed with 0.1% BSA/PBS twice, and prepared as a cell suspension of 1 × 10^7^ cells/50 µl in 0.1% BSA/PBS. Subsequently, the cell suspension was stained simultaneously with two monoclonal antibodies, anti-human CD44-APC (clone G44-26; BD Biosciences, San Diego, CA) and anti-human EpCAM-FITC (clone EBA-1; BD Biosciences), for 30 min on ice. APC- and FITC-labeled control isotypes with matched concentrations of the above two antibodies were used to set the gating levels. The stained cells were diluted to 2 ml in 0.1% BSA/PBS and centrifuged at 250 × *g* for 5 min. The supernatant was removed, and 0.5 ml of PBS and 40 ng of propidium iodide (PI) were added to the cell pellet. Flow-cytometric analysis was performed using a BD FACSaria cell sorter (Becton Dickinson, San Jose, CA). Forward and side scatter width profiles were used to eliminate cell doublets. Dead cells were eliminated by excluding PI-positive cells. The resulting single-cell populations were separated by four gates, followed by each antibody of fluorescence intensity at a range of 0–10^3^ (negative-low expression, low/−) and 10^3^–10^5^ (high expression, high). The sorted cells, CD44^high^/EpCAM^high^ or CD44^low/−^/EpCAM^high^ cells, were immediately washed with 0.1% BSA/PBS twice and cultured in 10% FBS/DMEM.

### Culture and colonosphere formation

HT-29 cells were trypsinized from culture plates, washed with PBS twice, suspended in stem cell medium (SCM) composed of DMEM/F12 medium, 20 ng/ml EGF, 10 ng/ml of bFGF, 0.2% of B27 and antibiotic-anti-mycotic agent, plated at a density of 3 × 10^4^ cells/ml SCM into 24-wells of ultra-low attachment plate (Corning, NY) and incubated for 5 days at 37°C in a humidified atmosphere containing 5% CO_2_. All experiments with Csps described below were performed using Csps grown for 5 days.

### Preparation of culture medium with FuOH

A total of 4 mM of FuOH reconstituted in tetrahydrofuran (THF) was added to 1% FBS/DMEM or SCM at a final concentration of 0.5 v/v%. After, the medium was filtered with 0.2 µm of cellulose acetate and a 1.0 ml aliquot was extracted with 4 ml of dichloromethane/methanol (1:1, v/v) and subjected to HPLC analysis. FuOH was analyzed by HPLC with a Waters 2695 Alliance200 system equipped with a photodiode array detector (Waters, Milford, MA) and a Shimadzu VP-ODS (250 × 4.6 mm i.d., 5 µm particle size) reversed-phase column. Column temperature and flow rate were 25°C and 1.0 ml/min, respectively. The monitoring wavelength and solvent mixture of FuOH were 450 nm and acetonitrile/methanol (45:55, v/v), respectively. The FuOH concentration was quantified from the peak area using a standard line.

### Cell proliferation

Flow cytometry-derived cells, CD44^high^/EpCAM^high^ and CD44^low/−^/EpCAM^high^ cells, after several passages, were seeded at a density of 5 × 10^4^ cells/ml in 10% FBS/DMEM into 12-well plates. After adherence for 5 h, the cells were reintroduced to 1% FBS/DMEM containing FuOH (0.1–2.0 µM) or vehicle (THF). After a 24-h incubation, WST-1 reagent was added to the medium and the cell viability was measured at 450 nm by using an ELISA reader (TECAN Japan, Tokyo, Japan). Additionally, 0.5 ml of SCM containing FuOH (0.1–5.0 µM) was applied to 0.5 ml of SCM of Csps cultured for 5 days in a 24-well of an ultra-low attachment plate and incubated for an additional 5 days. The medium was transferred to a microtube, centrifuged at 200 × *g* for 3 min, washed with PBS once, and suspended in 0.1 ml of SCM containing WST-1 reagent. The cell viability was measured at 450 nm by using an ELISA reader. For morphological analysis, Csps were stained with Hoechst 33342 (Sigma Aldrich, St. Louis, MO). Cellular and nuclear morphologies were observed by phase-contrast and fluorescence microscopy, respectively.

### Western blot

Akt (phosphorylation of Ser473 and pan), β-catenin (phosphorylation of Ser33/37/Thr41 and pan), cyclin D_1_, c-myc, NF-κB (p105 precursor and p50 active form) and PPARγ antibodies were purchased from Cell Signaling Technology (Danvers, MA). PPARδ was purchased from Santa Cruz Biotechnology (Santa Cruz, CA). β-Actin antibody was obtained from GeneTex (Irvine, CA). CD44 antibody was purchased from Thermo Fisher Scientific (Waltham, MA). EpCAM antibody was purchased from EXBIO (Praha, Czech). The cells sorted by flow cytometer were cultured in a normal 10-cm plate for several passages and collected. The epithelial type cells just before preparing Csps were used as the parental cells of the Csps. The Csps were seeded in a 10-cm ultra-low attachment plate at a density of 3 × 10^4^ cells/ml SCM, treated with FuOH and collected after 24 h. The cells were harvested, washed twice with PBS and then lysed in a lysis buffer for whole cell lysates. Fifty micrograms of whole cell proteins were separated on SDS-polyacrylamide minigels. The gels were then electroblotted onto a PVDF membrane. The PVDF was incubated in 5% BSA blocking buffer at room temperature and was probed with each of the primary antibodies in the blocking buffer following the manufacturer’s instructions overnight at 4°C. The membranes were washed and incubated with HRP-conjugated anti-mouse or anti-rabbit secondary antibodies. The membranes were washed and subsequently subjected to chemiluminescence reagents.

### Secondary colonosphere formation

HT-29 parental cells (PCs) and the Csps formed in SCM for 5 days were trypsinized from the culture plate, washed with PBS twice, suspended in SCM, seeded at a density of 3 × 10^4^ cells/ml SCM into 24 wells of an ultra-low attachment plate (Corning, NY) and incubated for 5 days at 37°C in a humidified atmosphere containing 5% CO_2_. After 5 days, the spheres derived from both populations were observed by phase-contrast microscopy. Cell growth of the primary and secondary Csps was assessed by WST-1 assay.

### Xenograft tumor experiments

Five-week-old male NOD-SCID (NOD.CB17-*Prkdc*^*scid*^/J) mice were purchased from Japan Charles River Laboratories (Kanagawa, Japan). The mice were housed in plastic cages with sterilized softwood chips as bedding in a barrier-sustained animal room at 24 ± 2°C and 55% humidity on a 12 h light/dark cycle. Food and water were available *ad libitum*. After a week of acclimation, the single cell suspension dissociated from the Csps was subcutaneously injected onto the backbone of all NOD-SCID mice at 3 × 10^4^ cells/100 µl 0.1% BSA/PBS using a 25.0-gauge 1 ml disposable syringe. The animals were observed daily for clinical signs and mortality. Tumor size and body weight were measured every 3 days for one month. The estimated tumor size was calculated by the formula (mm) × b^2^ (mm)/2 (a, long range: b, short range). NOD-SCID mice with tumors of roughly the same size were divided into two plastic cages of three mice each. FuOH/soybean oil was administered at 5 mg/kg body weight per mouse using a stomach sonde needle every 3–4 days for 2 weeks. The control group was given soybean oil only for each mouse. Tumor size and body weight were determined at the time of FuOH administration. The experiments were performed according to the “Guidelines for Animal Experiments in the National Cancer Center” and were approved by the Institutional Ethics Review Committee for Animal Experimentation in the National Cancer Center.

### Statistical analysis

All of the *in vitro* and *in vivo* experiments were performed at least twice and are presented as representative data. Significant differences between the means of two groups were determined by *t* test, and the differences were considered statistically significant when *p<0.05 and **p<0.01.

## Results

### Anti-proliferative effect of FuOH on CD44^high^/EpCAM^high^ cells

**Fig. 2:**
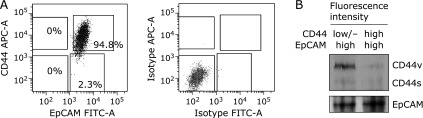
Flow cytometric isolation of colorectal cancer stem cell-like cells based on CD44 and EpCAM expression. (A) HT-29 cells were stained with CD44-APC and EpCAM-FITC monoclonal antibodies, analyzed and sorted by flow cytometer. The lines and gates in APC-FITC plots were used to sort the four cell populations. Fluorescence intensity of 0–10^3^ and 10^3^–10^5^ indicates negative or low expression (low/−) and high expression (high), respectively. Upper panel, plots of targeting cell markers. Lower panel, plots of isotype controls. (B) CD44 and EpCAM protein expression in the CD44^high^/EpCAM^high^ and CD44^low/−^/EpCAM^high^ cells detected by western blot.

**Fig. 3:**
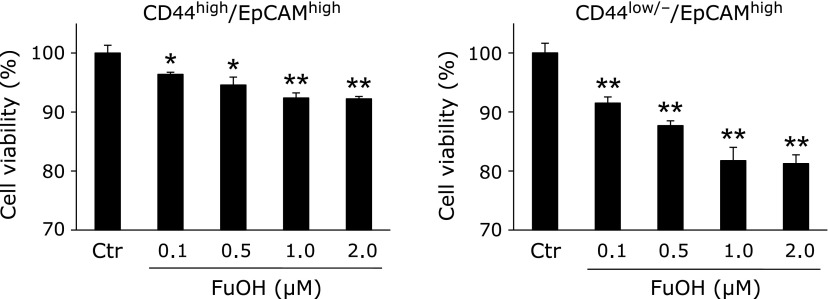
Anti-proliferative effects of fucoxanthinol (FuOH) on the CD44^high^/EpCAM^high^ and CD44^low/−^/EpCAM^high^ cells. CD44^high^/EpCAM^high^ and CD44^low/−^/EpCAM^high^ cells were incubated with 0.1–2.0 µM of FuOH for 24 h (1% FBS + DMEM). Cell viability was determined by the WST-1 assay. Values are means ± SE (*n* = 6). *p<0.05, **p<0.01 (*t* test, vs control).

The CD44 phenotype of HT-29 cells was presented as a 1,000-fold difference in expression intensity (APC-A, 10^2^–10^5^). Most cells were CD44^high^ cells, and only a few were CD44^low/−^ cells. The EpCAM phenotype was almost identical for the expression intensity (FITC-A, 10^3^–10^4^), and all phenotypes were EpCAM^high^ cells. EpCAM^low/−^ cells were not detected. A combined analysis of CD44 and EpCAM represented two different populations: CD44^high^/EpCAM^high^ cells (94.8%) and CD44^low/−^/EpCAM^high^ cells (2.3%). Therefore, these two populations were sorted by flow cytometry for the further experiments. The results of western blot analysis using the sorted cells were compatible with expression intensity of CD44 and EpCAM obtained from flow cytometry (Fig. 2B). Subsequently, the anti-proliferative capability of FuOH on CD44^high^/EpCAM^high^ and CD44^low/−^/EpCAM^high^ cells was examined. Treatment with 0.1–2.0 µM FuOH inhibited the growth of CD44^high^/EpCAM^high^ cells in a dose-dependent manner. However, the cells were less sensitive to the agent than the CD44^low/−^/EpCAM^high^ cells (Fig. 3). The cell viabilities were as follows: 0.1 µM FuOH, 96.4%; 0.5 µM, 94.6%; 1.0 µM, 92.4%; and 2.0 µM, 92.2% in CD44^high^/EpCAM^high^ cells; and 0.1 µM, 91.5%; 0.5 µM, 87.7%; 1.0 µM, 81.8%; and 2.0 µM, 81.2% in CD44^low/−^/EpCAM^high^ cells.

### Molecular characteristics of colonospheres

**Fig. 4:**
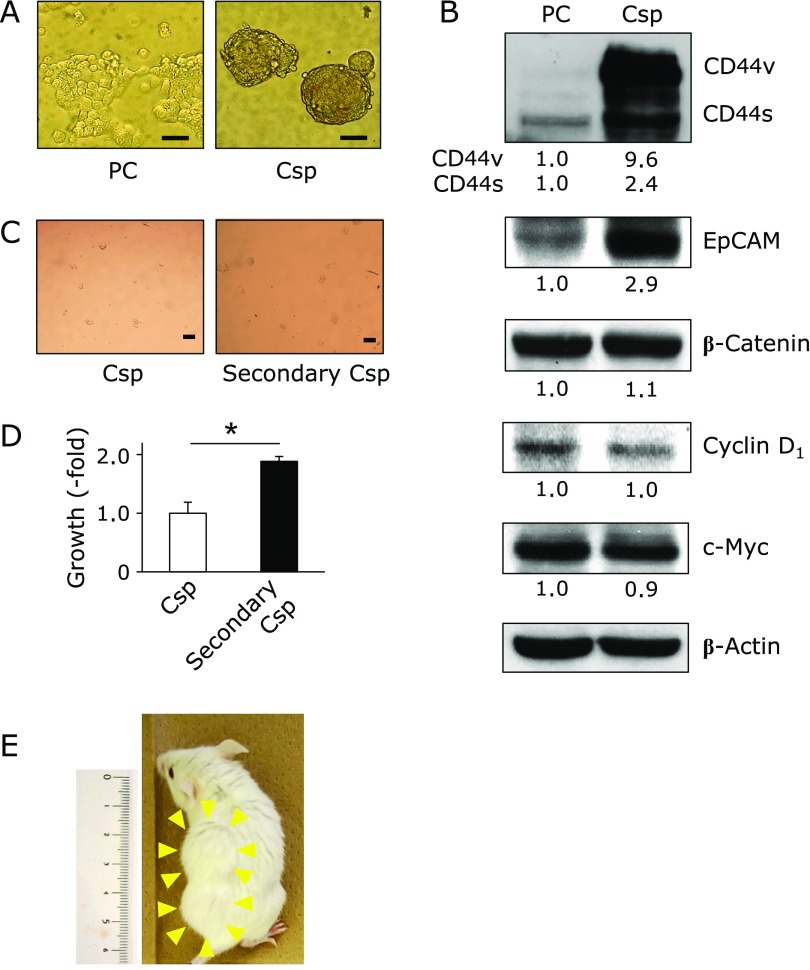
Stemness of colonospheres. Colonospheres (Csps) were formed with SCM for 5 days. (A) Photograph of PCs and Csps under phase-contrast microscopy. Bar, 100 µm. (B) The pre-treatment cells (parent cells, PCs) were collected and subjected to western blot. The value of each band was normalized to that of the β-actin band density from the image. (C) The primary Csps generated from HT-29 cells were dissociated into a single-cell suspension and subsequently subjected to secondary sphere formation. After 5 days, the spheres derived from both populations were observed under phase-contrast microscopy. (D) The cell growth of primary and secondary Csps was assessed by the WST-1 assay. Values are means ± SE (*n* = 6). *p<0.05, **p<0.01 (*t* test, vs control). (E) A typical subcutaneous tumor produced by an injection of Csps cells after 2 months. The implanted cells (3 × 10^4^ cells) were obtained from Csps as described in Materials and Methods.

The protein profiles of Csps and the PCs (Fig. 4A) were determined by western blot. The CD44 variant forms (CD44v), CD44 standard form (CD44s) and EpCAM were significantly overexpressed in Csp compared with PC. The expression of β-catenin, a key protein for activation of Wnt/β-catenin signals, could not discriminate between the Csps and PCs. Cyclin D_1_ and c-Myc, a representative cell cycle regulator and a tumor transcriptional factor, respectively, was not altered between the two cell types. In the next experiment, replication competence was examined in the Csp cells. More secondary Csps were observed than primary Csps (Fig. 4C). The cell viability of secondary Csps increased 1.9-fold compared to the primary Csps (Fig. 4D). After the NOD-SCID mice were implanted with a cell suspension of Csps (3 × 10^4^ cells), 80% of the mice developed a xenograft tumor within a month (Table 1 and Fig. 4E).

### Anti-proliferative effect of FuOH in colonospheres

**Fig. 5:**
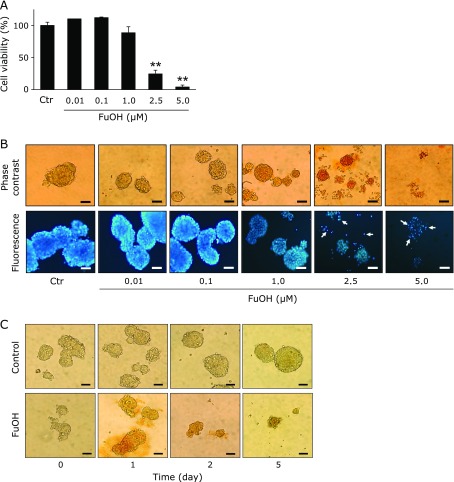
Anti-proliferative effects of fucoxanthinol (FuOH) on colonospheres. Csps formed with SCM for 5 days were treated with 0.01–5.0 µM of FuOH for 5 additional days under SCM. (A) Cell viability was determined by the WST-1 assay. Values are means ± SE (*n* = 3). **p<0.01 (*t* test vs control). (B) After the disintegration effect of FuOH on Csps was observed under phase-contrast microscopy, the cells were stained with Hoechst 33342. Arrows show the chromatin condensation and nuclear fragmentation of Csps cells under fluorescence microscopy. Bar, 100 µm.

Treatment with 2.5 and 5.0 µM FuOH inhibited the growth of Csps in a dose-dependent manner (Fig. 5A). The cell viabilities were as follows: 0.01 µM FuOH, 110.2%, 0.1 µM, 112.2%; 1.0 µM, 88.6%; 2.5 µM, 24.5%; and 5.0 µM, 4.1%. The IC_50_ value of FuOH against Csp was 1.8 µM. The vehicle (THF) alone showed no effect on cell proliferation. Moreover, treatment with 2.5 and 5.0 µM FuOH induced Csp disintegration, chromatin condensation and nuclear fragmentation, characteristics of apoptosis (Fig. 5B). At a dose of 5.0 µM, FuOH treatment inhibited cell growth of Csps in a time-dependent manner (Fig. 5C).

### Change of molecular expression in colonospheres by FuOH

**Fig. 6:**
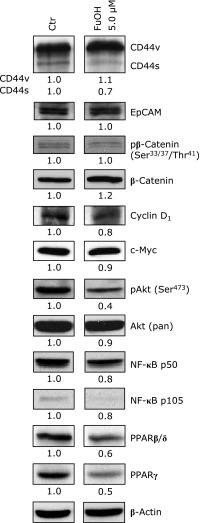
Western blot analysis of colonospheres with or without fucoxanthinol (FuOH) treatment. Csps formed with SCM for 5 days were treated with 5.0 µM of FuOH for 24 h. The control was used THF alone. These cells were collected and subjected to western blot. The amount of the protein was measured, and the same amount of protein was loaded. Moreover, the value of each band was normalized to that of the β-actin band density from the image.

To investigate the mechanism of FuOH-induced apoptosis in Csps, changes in the expression of the proteins involved in growth and inflammation were evaluated in Csps. Csps were treated with FuOH for 24 h (Fig. 6). FuOH treatment remarkably decreased the expression of pAkt (Ser^473^), an active form of Akt, PPARβ/δ, and PPARγ compared with non-treated controls. FuOH treatment did not change CD44v, CD44s, EpCAM, pβ-catenin (Ser^33/37^/Thr^41^), β-catenin, cyclin D_1_, Akt (pan), NF-κB p50, an active form of NF-κB, or NF-κB p105, a precursor form of NF-κB, compared with non-treated controls.

### Inhibition of xenograft tumor development by FuOH

**Fig. 7:**
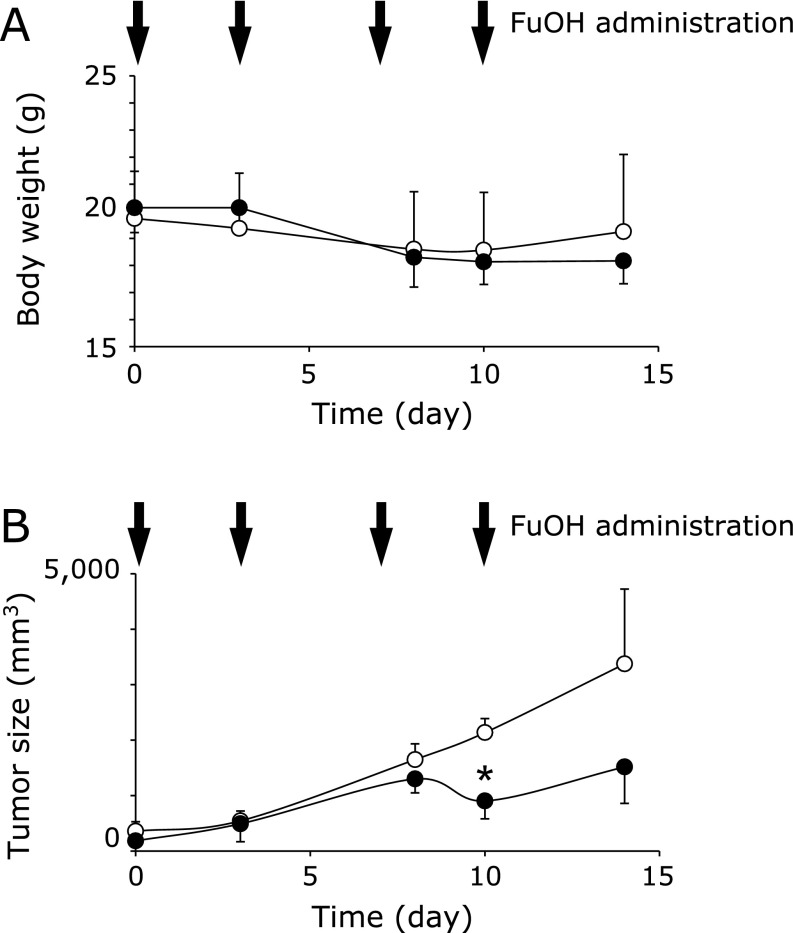
Csps-xenografted mice with or without fucoxanthinol (FuOH) administration. Csps were formed with SCM for 5 days. The single-cell suspension dissociated from Csps was injected subcutaneously on the back of the SCID mice at 3 × 10^4^ cells/100 µl (0.1% BSA/PBS). The SCID mice bearing similar sized tumors were divided into two groups (*n* = 3 mice each). FuOH (I) dissolved in soybean oil (100 µg/100 µl) was gavaged to mouse using a stomach sonde needle every 3–4 days for 2 weeks. The control group was given soybean oil alone (○). The body weight (A) and tumor size (B) were measured when given FuOH and at the end of the experiment.

NOD-SCID mice bearing similar sized tumors were divided into two plastic cages of three mice each. FuOH/soybean oil was administered at 5 mg/kg BW per mouse by gavage every 3–4 days for 2 weeks. Little difference in body weight was observed between the FuOH-treated group and the non-treated control group during the experimental periods (Fig. 7A). The tumor sizes were approximately the same between both groups until 8 days of treatment. FuOH significantly suppressed tumor size after 10 days of treatment (Fig. 7B).

## Discussion

Increasing evidence has recently suggested that CCSCs are largely responsible for colorectal carcinogenesis, cancer recurrence and cancer drug resistance. However, to date, little is known about the direct effect of dietary carotenoids on CCSCs. This is the first report demonstrating the inhibitory effects of FuOH on CCSCs.

In the current study, we first attempted to isolate the double-positive CD44 and EpCAM population of HT-29 cells using flow cytometry (Fig. 2). Consequently, the CD44^high^/EpCAM^high^ cells were separated as a large subset (94.8%) within HT-29 cells, and the CD44^low/−^/EpCAM^high^ cells were the minority subset (2.3%). CD44, a hyaluronan receptor, is known to promote tumor invasion and metastasis by binding to a pericellular matrix of hyaluronan in CRC cells.^(29,30)^ Moreover, CD44 expression is positively associated with colorectal carcinogenesis and with CRC stemness.^(31)^

FuOH significantly suppressed the growth of CD44^high^/EpCAM^high^ cells. CD44^high^/EpCAM^high^ cells are considered to be more chemoresistant than CD44^low/−^/EpCAM^high^ cells (Fig. 3). Therefore, we next evaluated whether FuOH effectively induces apoptosis in chemoresistant CCSCs. CD44 is a standard form of CD44s, and it has several variant forms (CD44v). The CD44v expressed in HT-29 cells is v3 or v6, and these two variants are involved in tumor formation, metastatic progression and/or chemoresistance in CRC.^(30,32–34)^ Hydrogen peroxide (H_2_O_2_) produced by xanthine/xanthin oxidase is suggested as an antiproliferative inducer in CRC cells.^(35)^ CD44v is reported to protect cells from such an antiproliferative inducer through interaction with xCT, a subunit of a cysteine-glutamate transporter. Therefore, chemoresistance observed in CCSC from HT-29 cells could be partly affected by CD44v expression state. In the present study, we did not compare the difference of anti-proliferative effects of FuOH between the expression of CD44s and CD44v in HT-29 cells. Further investigation is needed to determine whether FuOH differently affects CRC cells expressing different CD44 isoforms.

We also examined the stemness characteristics of CCSC by assessing Csps formation from HT-29 cells (Fig. 4). The Csps from HT-29 cells had CCSC-like properties, such as overexpression of CCSC markers, Csp formation and Csp-reformation activities and tumorigenesis. FuOH inhibited proliferation and induced apoptosis in the cells of Csps (Fig. 5). FuOH strongly suppressed the expression of pAkt (Ser^473^), an active form of Akt, PPARβ/δ, and PPARγ (Fig. 6). These three molecules are all involved in transactivation of cyclooxygenase-2 (COX-2), a key factor in colon carcinogenesis. Previously, we demonstrated that sesamol, a natural polar lipid as well as FuOH, suppressed COX-2 transcriptional activity in CRC cells and the mRNA expression in the intestinal polyp part in* Apc*^Min/+^ mice.^(36)^ The COX-2 transactivation could also play some roles in FuOH-induced apoptosis in the cells of Csp. Both PPARγ and PPARδ nuclear receptors are assumed to promote the growth of both normal colonic crypt cells and CRC.^(37,38)^ PPARγ down-regulation induced by antagonist- or RNA interference causes apoptosis via reduction of cellular adhesion molecule expression.^(39)^ We recently found that FuOH induced anchorage-dependent apoptosis, anoikis, through modification of integrin signals in adherent human CRC cells (data not shown). It is known that attenuation of integrin results in inhibition of FAK activation following the inhibition of several signal transduction pathways, such as PI3K/Akt, MAPK, NF-κB and PPARγ. Therefore, FuOH might induce anoikis in Csps as well as in adherent CRC cells. Fx has been reported to inhibit the cell cycle, caspase activation, mitochondrial membrane potential, and mammalian replicative DNA polymerase activity in human cancer cells.^(13–19)^ These molecular alterations may contribute to the apoptosis induction of Csps by FuOH administration.

FuOH significantly suppressed tumor development of a cell suspension obtained from Csps (Fig. 7B). Intake of dietary brown algae delivers some Fx metabolites into the blood and various tissues.^(20)^ With dietary intervention of wakame (6.1 mg Fx/day) for 1 week, FuOH was detected at a level of <1 nmol/L in human plasma levels.^(20)^ A single-dose of oral administration of the extract (31 mg Fx) of brown algae (kombu) in healthy volunteers increased plasma level of FuOH (44.2 nmol/L).^(21)^ The clearance of FuOH in human blood is fast: the half-life of FuOH is reported to be 4 h.^(21)^ Two weeks of supplementation (200 mg Fx/kg diet) to mice accumulates as three main metabolites, FuOH, Amx A and *cis*-Amx A, in various tissues at higher concentrations than the plasma, at approximately 1 µM of total Fx metabolites.^(40)^ Therefore, in this study, sufficient amounts of FuOH could reach in the tissues where Csps were injected to inhibit tumor development. β-carotene induces CSC differentiation in neuroblastomas through ERK activation and down-regulation of Drosophila delta-like 1 homolog.^(41)^ One of the apocarotenoids, crocetinic acid, a compound from saffron, suppresses cell growth in human pancreatic CSCs through inhibiting Sonic Hedgehog and EGFR.^(42)^

In conclusion, FuOH showed distinct anti-proliferative effects on two CCSC-like cell populations, CD44^high^/EpCAM^high^ cells and Csps. FuOH suppressed key molecules responsible for cancer survival and tumorigenesis in Csps, such as pAkt (Ser^473^), PPARβ/δ, and PPARγ. Our findings suggest that FuOH could be used as a chemopreventive agent against human CCSC.

## Figures and Tables

**Table 1:** Tumor formation in the NOD/SCID mice.

Time (day)^a^	Body weight (g)	Incidence of tumor (%)^b^	Tumor volume (mm^3^)^c^
3	18.9 ± 1.3^d^	0	0
13	20.4 ± 1.1	0	0
20	20.9 ± 1.2	20	75.0 ± 17.7
24	20.8 ± 1.2	40	655.0 ± 271.4
31	20.8 ± 1.4	80	550.3 ± 410.4

^a^Days counted after injection of single cell suspension from colonosphere. ^b^The percentage per ten of mouse. ^c^The estimated tumor size were calculated by the fomula of a × b^2^/2; a, long range; b, short range. ^d^Mean ± SD.

## Abbreviations

CCSC: colorectal cancer stem cell
FuOH: fucoxanthinol
Fx: fucoxanthin
